# Supplementary material for: Moral luck in investment contexts: We consciously find unprofitable investments less moral
Source: PLoS One. 2023 Jan 17;18(1):e0278677. doi: 10.1371/journal.pone.0278677 (PMC9844880; doi:10.1371/journal.pone.0278677)
Supplement: S3 Data — (DOCX) [file pone.0278677.s003.docx]

# S3 Appendix. Vignettes

The six different vignettes read as follows:

*BB: Amanda Roberts is the Director of Financial Planning and Analysis of a large bank. Mrs. Roberts is responsible for the financial planning of the bank and has the goal to increase the financial assets. Several years ago, after consulting with financial advisors, she decided to buy complex financial products whose price development depends on the fluctuations of the oil price. Due to a significant oil price change in the following years, the bank recently had to accept a strong loss.*

*BG: Amanda Roberts is the Director of Financial Planning and Analysis of a large bank. Mrs. Roberts is responsible for the financial planning of the bank and has the goal to increase the financial assets. Several years ago, after consulting with financial advisors, she decided to buy complex financial products whose price development depends on the fluctuations of the oil price. Due to a significant oil price change in the following years, the bank recently recorded a large profit.*

*CB: Amanda Roberts is the Director of Financial Planning and Analysis of an automotive company. Mrs. Roberts is responsible for the financial planning of the company and has the goal to increase the financial assets. Several years ago, after consulting with financial advisors, she decided to buy complex financial products whose price development depends on the fluctuations of the oil price. Due to a significant oil price change in the following years, the company recently had to accept a strong loss.*

*CG: Amanda Roberts is the Director of Financial Planning and Analysis of an automotive company. Mrs. Roberts is responsible for the financial planning of the company and has the goal to increase the financial assets. Several years ago, after consulting with financial advisors, she decided to buy complex financial products whose price development depends on the fluctuations of the oil price. Due to a significant oil price change in the following years, the company recently recorded a large profit.*

*MB: Amanda Roberts is the Director of Financial Planning and Analysis of a municipal administration. Mrs. Roberts is responsible for the financial planning of the administration and has the goal to increase the financial assets. Several years ago, after consulting with financial advisors, she decided to buy complex financial products whose price development depends on the fluctuations of the oil price. Due to a significant oil price change in the following years, the administration recently had to accept a strong loss.*

*MG: Amanda Roberts is the Director of Financial Planning and Analysis of a municipal administration. Mrs. Roberts is responsible for the financial planning of the administration and has the goal to increase the financial assets. Several years ago, after consulting with financial advisors, she decided to buy complex financial products whose price development depends on the fluctuations of the oil price. Due to a significant oil price change in the following years, the administration recently recorded a large profit.*
