# Supplementary material for: Moral luck in investment contexts: We consciously find unprofitable investments less moral
Source: PLoS One. 2023 Jan 17;18(1):e0278677. doi: 10.1371/journal.pone.0278677 (PMC9844880; doi:10.1371/journal.pone.0278677)
Supplement: Attachment — Submitted filename: 01d_Answer_to_Reviewer_3.pdf [file pone.0278677.s004.pdf]

Reviewer #3:

Thank you very much for the constructive comments on our manuscript. We think that our manuscript has very much benefitted from incorporating the suggestions you made in your referee report. In what follows, we will address each of your comments individually. Reviewer comments are highlighted in bold type. Below that, you will find our comments in italics.

In study 1, they found people rate higher immoral scores for a "loss" scenario than a "profit" scenario, similarly there are less people who believe it is an investment in the "loss" scenario" than the "profit scenario". Study 2 was to analyze whether the observed pattern in Study 1 is based on moral reasoning or an unreflected moral intuition. 1. The literature has already showed people exhibit outcome bias (related to moral luck). I think the authors really need to emphasize how their work contribute to the literature. For instance, for study 1, even without their study, at least some readers would already think that people would base their evaluations and interpretations on outcome.

This objection of Reviewer 3 is justified. We have tried to supplement and enhance the argumentation with some comments at the mentioned point.

2. The section on "the concept of moral luck" is a bit too long and nearly included an entire paragraph of the original words from Kant. I don't think it is necessary. It would make the story to stay on track better.

We thank Reviewer 3 for this objection and have removed the lengthy quote from Kant at this point.

3. Figure 2a appear to show the results after they see the outcome. I think readers would be interested to see how their ratings are immediately after they see only the decision but before the outcome is revealed.

We now report these numbers and conduct further testing. The information requested here by Reviewer 3 is now shown in the paper.

4. Figure information can be better presented. For instance, the authors can easily include a legend name of what each color of the bar represent. The authors could also consider showing the p-values on the graph (or the show the ** on the graph). Moreover, please provide a name for the figure, at the first glance Figure 1a and Figure 2a appear to be the same.

We have revised the graphs as requested and do now provide figure names.
