# Supplementary material for: Moral luck in investment contexts: We consciously find unprofitable investments less moral
Source: PLoS One. 2023 Jan 17;18(1):e0278677. doi: 10.1371/journal.pone.0278677 (PMC9844880; doi:10.1371/journal.pone.0278677)
Supplement: Attachment — Submitted filename: 01b_Response_Reviewer1_minor_revision.docx [file pone.0278677.s005.docx]

Reviewer #1:

Thank you very much for the constructive comments on our manuscript. We think that our manuscript has very much benefitted from incorporating the suggestions you made in your referee report. In what follows, we will address each of your comments individually.

**⁃ definition of “moral luck”: I guess the authors have been trying to rephrase Nelkin’s sentence “Moral luck occurs when an agent can be correctly treated as an object of moral judgment despite the fact that a significant aspect of what she is assessed for depends on factors beyond her control.” Nelkin states that the agent is *being judged*. In the manuscript, the authors give the impression that it is the agent who is *making a judgement*. This may not be intended but readers can easily get this impression. Please revise.**

*We rephrased the respective sentence to avoid the misunderstanding described by the reviewer.*

**⁃ There is still no rationale given for the chosen sample sizes. Please add these.**

*We added information on a power analysis in footnote 1 of the revised version of the paper.*

**⁃ Study 2: I do not understand why the “change score” removes the worry about initial differences in pre-evaluations between groups. Please add information about these.**

*We added footnotes 3 and 5 to provide the required information which shows that initial differences in pre-evaluations and pre-labels were not statistically significant.*

**⁃ Please add tables for one-way ANOVAs for both studies for sake of completeness**

*Tables for one-way ANOVAs have been added to Study 1 and Study 2.*

**⁃ As far as I see, there is still no main effect of outcome in Study 2. Please acknowledge this.**

*As per request of reviewer 2, we do now focus on the change score as our dependent variable in Study 2. As our new ANOVAs show, there is a main effect of outcome on the change score. There is, however, still no main effect of outcome on the label change of the investment. We now acknowledge this in the limitations of our study.*

**⁃ Williams reference: Thanks, now I see where the talk of moral luck as an oxymoron is coming from. Williams (“Moral Luck”, 1981) is a locus classicus for the term and Williams is generally regarded as being supportive of moral luck, so the way the term is introduced currently gives the misleading impression that Williams thought moral luck was an oxymoron. Instead, he wanted to draw attention to a puzzle. Please adjust this.**

*Thank you for pointing this out. We rephrased the respective passage to make this clear now.*

**⁃ Tables 1 and 2 contain three sub-columns (under “profit”) for “bank”, shouldn’t two of these be “company” and “municipality”?**

*Thank you for pointing this out. The table was changed according to a request by Reviewer 2.*
