# Supplementary material for: Moral luck in investment contexts: We consciously find unprofitable investments less moral
Source: PLoS One. 2023 Jan 17;18(1):e0278677. doi: 10.1371/journal.pone.0278677 (PMC9844880; doi:10.1371/journal.pone.0278677)
Supplement: Attachment — Submitted filename: 01b_Letter_to_Reviewer_minor_revision2_Reviewer3.docx [file pone.0278677.s006.docx]

Reviewer #3:

We are grateful for Reviewer 3’s careful reading of our manuscript and apologize that we did not address all concerns in the previous revision of our manuscript. We are confident that the current revision takes care of the reviewer’s remaining concerns. In what follows, we address each remaining concern individually.

**Reviewer #3: The authors really need to read their paper before their submission, in this submission, Table 2 has a table that is completely empty. And the table below it is also broken by pages.**

*ANSWER: We are very sorry for this mistake. We have deleted the empty table and corrected the broken table.*

***In one of my comment (maybe I said Table 4, but it is Table 5 this time) it did show "outcome x order" has a significant effect. I don't think the authors have addressed my comments and barely responded to it. I read what is on their table. If it was a misunderstanding, then the authors need to clarify it in in depth.***

*ANSWER: We apologize for not having addressed this comment sufficiently. In the revised version of the manuscript, we follow the previous suggestion of Reviewer 3 and perform an additional analysis that focuses on the condition where the labeling of the decision was done first. This analysis is now summarized in Table 6 which is discussed on p. 15 of the revised version of the manuscript. It indeed turns out that the influence of the investment’s outcome no longer predicts the labeling of the decision, if only those respondents are considered who label the investment before it is evaluated.*

***Moreover, the tables generally can be formatted a bit better (please refer to some other publications either on PLOS ONE or other journals).***

***- e.g. spaces between figures/tables and text do not appear to follow any systematic pattern (e.g. see those above Figure 1a's title)***

***- tables just generally appear to be a bit like raw presentation. Some of the boarders looks thicker while others are thinner.***

*ANSWER: We referred to other PLOS ONE publications where the tables have an upper and a lower frame as well as inner frames. Tables are now formatted consistently and spaces between figures and tables have been checked for consistency as well.*
